# The Relationship between Insect Resistance and Tree Age of Transgenic Triploid *Populus tomentosa* Plants

**DOI:** 10.3389/fpls.2018.00053

**Published:** 2018-01-26

**Authors:** Yachao Ren, Jun Zhang, Guiying Wang, Xiaojie Liu, Li Li, Jinmao Wang, Minsheng Yang

**Affiliations:** ^1^Forest Department, Forestry College, Hebei Agricultural University, Baoding, China; ^2^Hebei Key Laboratory for Tree Genetic Resources and Forest Protection, Baoding, China; ^3^Langfang Academy of Agriculture and Forestry Sciences, Langfang, China

**Keywords:** *Populus tomentosa*, transgenic lines, toxicity, mortality rate, correlation analysis

## Abstract

To explore the stability of insect resistance during the development of transgenic insect-resistant trees, this study investigated how insect resistance changes as transgenic trees age. We selected 19 transgenic insect-resistant triploid *Populus tomentosa* lines as plant material. The presence of exogenous genes and Cry1Ac protein expression were verified using polymerase chain reaction (PCR) and enzyme-linked immunosorbent assay (ELISA) analyses. The toxicity for *Clostera anachoreta* and *Lymantria dispar* was evaluated by feeding fresh leaves to first instar larvae after the trees were planted in the field for 2 years and after the sixth year. Results of PCR showed that the exogenous genes had a long-term presence in the poplar genome. ELISA analyses showed significant differences existed on the 6-year-old transgenic lines. The insect-feeding experiment demonstrated significant differences in the mortality rates of *C. anachoreta* and *L. dispar* among different transgenic lines. The average corrected mortality rates of *C. anachoreta* and *L. dispar* ranged from 5.6–98.7% to 35.4–7.2% respectively. The larval mortality rates differed significantly between the lines at different ages. Up to 52.6% of 1-year-old transgenic lines and 42.1% of 2-year-old transgenic lines caused *C. anachoreta* larval mortality rates to exceed 80%, whereas only 26.3% of the 6-year-old transgenic lines. The mortality rates of *L. dispar* exhibited the same trend: 89.5% of 1-year-old transgenic lines and 84.2% of 2-year-old transgenic lines caused *L. dispar* larval mortality rates to exceed 80%; this number decreased to 63.2% for the 6-year-old plants. The proportion of 6-year-old trees with over 80% larval mortality rates was clearly lower than that of the younger trees. The death distribution of *C. anachoreta* in different developmental stages also showed the larvae that fed on the leaves of 1-year-old trees were killed mostly during L_1_ and L_2_ stages, whereas the proportion of larvae that died in L_3_ and L_4_ stages was significantly increased when fed on leaves of 6-year-old trees. Results of correlation analysis showed there was a significant correlation between the larvae mortality rates of trees at different ages, as well as between Cry1Ac protein contents and larvae mortality rates of 6-year-old trees.

## Introduction

Poplars are trees commonly used in afforestation programs and are widely planted throughout most of China for various purposes. Their high growth rate and usefulness in the timber and pulpwood industries, shelterbelt creation, and plantation development make poplars the most important tree for afforestation (Wang et al., 2012). Over the past 30 years, China has afforested more than 54 million hectares of land and become the country with the greatest amount of afforestation in the world (Hui, 2009). In poplar plantations, the use of a single or a few clones with low genetic diversity has led to increasing insect pest problems. Insect damage is often caused by commonly occurring lepidopterans, such as *Clostera anachoreta* Fabricius (Lepidoptera, Notodontidae), *Lymantria dispar* Linn (Lepidoptera, Lymantriidae), and *Hyphantria cunea* Drury (Liu et al., 2011). Insect pests have severely damaged and even killed large swathes of trees in some areas. Thus, there is an urgent need to cultivate insect-resistant varieties. Genetic engineering has great potential compared with conventional breeding methods and has already made great gains in the transformation of poplar varieties (Génissel et al., 2003; Buonamici et al., 2011).

Studies on the cultivation of insect-resistant transgenic poplars have been underway since 1983. Nearly 20 poplar species exhibit insect resistance through genetic engineering (Fladung and Ewald, 2006). The *Cry1Ac* gene from the bacterium *Bacillus thuringiensis* (*Bt*) confers specific resistance against several lepidopteran species. A *Cry1A* endotoxin gene was initially used to create a transgenic hybrid poplar in 1991 (McCown et al., 1991). Transgenic trees showed resistance to a variety of insects in the follow-up study (Ramachandran et al., 1993; Robinson et al., 1994). In China, *Populus nigra* was successfully modified using *Cry1A*c in 1993. Three plants were selected from 54 regenerated transgenic plantlets according to plant height, leaf shape, leaf color, and insect-resistance (Tian et al., 1993). This transgenic poplar was authorized for commercial production in 2002 (Lu and Hu, 2006). Several poplars modified using the *Cry1Ac* gene were developed in this study and exhibited toxicity against lepidopteran larvae in laboratory tests (Chen et al., 1995; Wang et al., 1997).

Plant proteinase inhibitors also play important roles in flora defense systems against insects and pathogens. Transgenic poplar trees that expressed a potato trypsin inhibitor gene (*PIN-II*) were reported in another study in 1987 (McNabb, 1987). *Sagittaria sagittiflia* var. *sinensis*, commonly known as “arrowhead,” is a perennial aquatic herb and tuber. Two arrowhead proteinase inhibitors have been identified, namely, API-A and API-B. These inhibitors inhibit some digestive enzymes in the intestinal canal of insects, thus causing anorexia and subsequently the inhibition of the growth and development of the insects (Lawrence and Koundal, 2002; Zhang et al., 2005). Tian et al. constructed a binary expression vector by using a partially modified *Cry1Ac* gene and the *API-A* gene and successfully introduced the genes into 741 hybrid poplars. Some selected lines showed high resistance to *C. anachoreta, L. dispar, H. cunea*, and other lepidopterans (Tian et al., 2000). This poplar was also authorized for commercial production in 2002 (Lu and Hu, 2006). The combination of the *Cry1Ac* and *API-A* genes was later utilized in translational studies involving various poplar species, such as *Populus tomentosa* and the Euramerican poplar (Li et al., 2007; Yang et al., 2012).

To date, studies on insect-resistant poplars have already been conducted and the resultant varieties have been successfully tested against several types of insect pests (Pi et al., 2012). However, most studies on transgenic insect-resistant poplars are conducted on young seedlings under laboratory or greenhouse conditions, and few studies have focused on the performance of adult transgenic *Bt* poplars under field conditions. Hu et al. (2007) investigated the larvae density of *Bt*-transformed *P. nigra* planted in Xinjiang province, China, during 1997–2001. The larvae density in the soil decreased from 18 heads m^−2^ in 1997 to 8 heads m^−2^ in 2001, but the larvae density increased in the non-transgenic plantation. They also found that the leaf loss caused by *Apocheimia cinerarius* Erschoff in the *Bt*-transformed plantation in Huairou, Beijing, was less than 20%, whereas it was 90% in the non-transgenic poplar plantation. However, for different transgenic lines, no information was given on changes in resistance over time. Other field studies on *Bt* transgenic poplars focused on safety evaluation; investigations were mostly conducted on the influence of insect species and the quantity of target and non-target insects (Gao et al., 2003; Zhang et al., 2004; Jiang et al., 2009). Few studies focused on different transgenic lines and how their insect resistance changed as the trees aged.

The presence and efficacy of exogenous genes in the transgenic forest, the stability of exogenous gene expression, and the effects of the natural environment and plant growth and development on the exogenous genes remain unclear. The inheritance and expression stability of exogenous genes in transgenic trees are important factors in the application of genetic engineering technology (Ye et al., 2011). The asexual propagation of transgenic trees involves a series of somatic cell divisions and propagations. Somatic cell division and propagation may result in the loss of exogenous genes (Jin et al., 2005). We studied the insect resistance changes of different lines transformed with *Bt* gene and *API* gene in different developmental stages to explore the exogenous gene expression differences and stability in adult transgenic poplar trees. Data from this study are essential to determine which changes in trees are caused by field conditions and to provide a basis for the selection of clones with high and stable insect resistance.

## Materials and methods

### Genetic engineering of poplar plants

**Figure 1:**
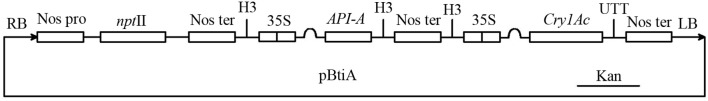
Construction of the pBtiA plant expression vector containing *API-A* and *Cry1Ac* genes. Nos pro, promoter of neomycin phosphotransferase gene; *npt*II, neomycin phosphotransferase gene; Nos ter, terminator of neomycin phosphotransferase gene; 35S, cauliflower mosaic virus 35S promoter; *API-A*, arrowhead proteinase inhibitor gene; *Cry1Ac, Bacillus thuringiensis* insecticidal crystal protein gene (Tian et al., 2000).

In the 1980s, professor Zhiti Zhu of Beijing Forestry University adopted sexual hybridization and chromosome-doubling technology to breed new varieties of poplars, including the triploid *P. tomentosa* (*P. tomentosa* Carr. × *P. bolleana* Louche × *P. tomentosa* Carr.). This new variety had high material quality, high pulp yields, fast growth, and a short rotation period (5 years) for the production of biomass for bioenergy and fiber use (Zhu et al., 1995). The plant transformation vector PBtiA contains a partly modified *Cry1Ac* gene and the arrowhead proteinase inhibitor *API-A* gene was created in 2000 (Figure 1). In this vector, neomycin phosphotransferase (*npt*II) was the selectable marker gene (Tian et al., 2000). The PBtiA vector was used to transform triploid *P. tomentosa* via the *Agrobacterium tumefaciens-*mediated transformation method with the aim of creating a new insect-resistant poplar. Fifty regenerated lines (named lines 1–50) with kanamycin resistance were obtained (Yang et al., 2006). Leaves from 1-year-old field plants were analyzed via polymerase chain reaction (PCR) amplification and enzyme-linked immunosorbent assay (ELISA) tests. The incorporation of the exogenous genes into the genome of the transgenic lines was confirmed using Southern blot and Western blot analyses (Yang et al., 2006; Wang et al., 2007).

### Plant material

In this study, 19 transgenic lines (lines 1, 2, 6, 7, 8, 10, 11, 16, 17, 21, 22, 24, 26, 29, 31, 41, 45, 46, and 47) were randomly selected from the above 50 lines and were used as materials for our experiment. Non-transgenic plants were used as controls. Four lines 7, 10, 11, and 21 were selected for Southern blot detection, the results showed that the exogenous genes in poplar genome were single copy (Yang et al., 2006). Transgenic and control trees were planted on nursery land that was used specifically for planting transgenic poplars, as approved by the State Forestry Administration. The testing field (38°83′N, 115°45′E) was characterized by flat terrain and plenty of natural light. We adopted a randomized complete block design (including 19 transgenic lines and control) for the field trial which contained two plants in each plot, repeated three times. All trees were planted at a spacing of 1 m within rows and 2 m between rows. We examined trees of the same line at three different times when the trees were 1, 2, and 6 years old.

### PCR molecular analysis

In this study, the presence of the *Cry1Ac* gene and the *npt*II gene in the 19 transgenic plants was analyzed using PCR to determine whether the genome retained the exogenous genes. The DNA of each transgenic line was extracted from 100 mg of mature leaves collected from field plants. DNA extraction was performed using the cetyltrimethyl ammonium bromide (CTAB) method (Sambrook et al., 1990, 1992). The DNA was eluted using 100 μL of TE at 65°C for 10 min and then stored at −20°C.

PCR analysis of the genomic DNA was performed using the oligonucleotide primers 5′-CTGACGTAAGGATGACGCAC-3′ and 5′-ACTATTGATAGTCGCGGCATC-3′ to detect the *Cry1Ac* gene. The DNA sample (20 μL) was amplified using 30 cycles of amplification (94°C for 50 s, 54°C for 100 s, and 72°C for 100 s). Final extension was at 72°C for 7 min and at 25°C for 1 min. The PCR product was 749 bp long. The *npt*II selectable marker gene was detected simultaneously using the primers 5′-CGGCGATACCGTAAAGCACC-3′ and 5′-GTCACTGAAGCGGGAAGGG-3′. The cycling parameters were as follows: 94°C for 50 s, 57°C for 50 s, and 72°C for 50 s for 30 cycles. The final extension steps were carried out at 72°C for 7 min and at 25°C for 1 min. The length of the PCR product of the *npt*II gene was 500 bp. The product was visualized with ethidium bromide after electrophoretic separation. Plasmid pBtiA was used as the positive control (CK^+^) and non-transgenic poplar DNA was used as the negative control (CK^−^).

### ELISA detection of the Cry1Ac protein

An ELISA kit for Cry1Ab/Cry1Ac (Agdia Co., USA) was used to determine Cry1Ac protein levels in the leaves. Three plants of each transgenic line were randomly selected from the experimental forest, and mature leaves were collected from the current central crown twigs to determine the toxin protein content. Leaves were ground to a fine powder in liquid nitrogen. Samples of 50 mg were used for protein extraction, and a series of analyses using ELISA were performed according to the manufacturer's instructions. The initial ELISA assay was performed on seven randomly selected 1-year-old lines (lines 5, 6, 9, 24, 27, 30, and 47). The levels of toxin protein ranged from 0.0011 to 0.0161 ng Cry1Ac protein/μg of the total leaf protein (Wang et al., 2007). The ELISA test was also performed on 6-year-old trees from the selected 19 lines (three lines of the first ELISA were included). All of the tests were repeated three times.

### Insects and insect bioassays

To determine the differential expression and stability of the exogenous genes in mature transgenic poplar trees, we studied the transgenic *P. tomentosa* lines with the exogenous genes at different developmental stages in terms of toxin gene stability and insect resistance. All transgenic plants were subjected to natural insect pressure in the fields. To avoid possible tolerance in insects, unhatched egg masses of the tested insects were collected from a non-genetically modified forest located in Shenzhou City, Hebei Province, 37°92′N, 115°53′ E. No genetically modified trees or other transgenic crops (such as cotton or soybean) were found in the area. The area was 150 km away from the transgenic nursery land. In April of the experiment year, unhatched egg masses were placed in a ventilated Erlenmeyer flask and covered. The eggs were allowed to hatch naturally at room temperature (18–20°C). The newly hatched larvae were used in the experiment.

Toxicity evaluation of the transgenic lines was performed on *C. anachoreta* and *L. dispar* first instar larvae when the trees were 1, 2, and 6 years old. Each experiment included 30 first instar larvae randomly collected from the egg masses. This test procedure was repeated three times for 19 transgenic lines in each experimental year. For each replicate and event, the insects were supplied with freshly detached leaves from the field-grown transgenic and control poplar plants. Mature poplar leaves were taken from the annual long middle branches in the middle of the crown. The leaf stalks were inserted into the flower mud (it is in the shape of a cube with the length of side is about 1.5 cm) in the flask to retain water. Jars (12 cm in height; 8.5 cm in diameter; and sealed with a nylon net) were used as containers with 30 larvae inside, and an 80-mesh nylon net was used to cover the opening. Leaves from the same transgenic line and non-transgenic control plants were replaced every other day.

To determine the insect death distribution at the different developmental stages (L_1_, L_2_, L_3_, and L_4_), further study on 1- and 6-year-old trees was conducted to determine the insect death distribution among the different developmental stages using *C. anachoreta* as an example. The insect developmental stage and larvae ecdysis were observed daily throughout the experiment. The death and mortality rates at the different developmental stages (L_1_, L_2_, L_3_, and L_4_) were recorded separately. The total mortality rates were calculated at the end (the 21st day) of each experiment. Toxicity evaluation of the transgenic lines started with the first instar (L_1_). The different instar insects were characterized as follows: Instar larvae L_1_; larvae were normally used 20 h after appearing from the egg block depending on the species. Other larval stages (L_2_, L_3_, and L_4_) were dependent on the insect species and characterized by their stage of molting.

### Statistical analyses

DPS Data Processing System (DPS 7.05) (Tang, 2010) and Microsoft Excel 2003 software were used for statistical analysis. The average mortality rates of the three replicates, the corrected mortality rates of each transgenic line, and the death distribution of *C. anachoreta* in different developmental stages (L_1_, L_2_, L_3_, and L_4_) were calculated and analyzed.

Correlation analyses: The correlation coefficients between 1- and 2-year-old trees, 1- and 6-year-old trees, and 2- and 6-year-old trees were analyzed. The correlation between the corrected mortality rate and Cry1Ac protein expression for the 6-year-old trees was also analyzed.

Variance and multiple comparisons analysis: The Cry1Ac protein expression variance and multiple comparisons in the 6-year-old transgenic plants were analyzed by one-way analysis of variance (ANOVA), followed by a Duncan's multiple range test; *P* < 0.05 was considered significant (Duncan, 1995). The 3-year averaged corrected mortality rates of the two types of insect species for each transgenic line were calculated, the differences between the 19 lines were analyzed by ANOVA, and *post-hoc* analysis was performed with Duncan's multiple range test.

$$\begin{array}{l} \;L_{n}\;larvae\;mortality\;rates\;=\;\frac{Number\;of\;dead\;larvae\;in\;L_{n}}{Initial\;number}\times 100\%\\ \;Total\;mortality\;rates\;=\;\frac{Total\;number\;of\;dead\;larvae\;at\;the\;end}{Initial\;number}\times 100\%\\ Average\;mortality\;rates\;of\;three\;years\;=\;\frac{Total\;mortality\;rates\;of\;three\;years}{3}\\ \;Corrected\;mortality\;rates\;=\;\frac{\left(Total\;mortality\;rates\;of\;transgenic\;plants\;-\;Total\;mortality\;rates\;of\;non\text{-}transgenic\;plants\right)}{\left(1\;-\;Total\;mortality\;rates\;of\;non\text{-}transgenic\;plants\right)}\times 100\% \end{array}$$

## Results

### PCR analysis of transgenic poplar plants

**Figure 2:**
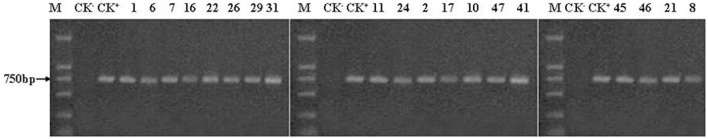
PCR amplification of the *Cry1Ac* gene. M, DL marker 2,000; CK^−^, negative control (non-transgenic poplar); CK^+^, positive control (plasmid pBtiA); the others are the 6-year-old transgenic lines. 19 transgenic lines and the positive control were all detected the expected bands (749 bp), while the negative control was not detected.

**Figure 3:**
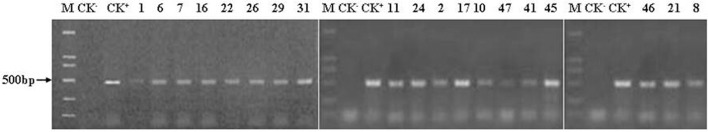
PCR amplification of the *npt*II gene. M, DL marker 2 000; CK^−^m negative control (non-transgenic poplar); CK^+^, positive control (plasmid pBtiA); the others are the 6-year-old transgenic lines. 19 transgenic lines and the positive control were all detected the expected bands (500 bp), while the negative control was not detected.

We conducted a PCR analysis of the 19 transgenic poplar plants and the control plants in three different years (at one, two, and 6 years of age). All analyses showed positive results with the expected bands except for the non-transgenic control plants. Given the large size of the PCR detection chart, only the PCR results of the 6-year-old trees are shown in Figures 2, 3 (others not shown here).

The amplified *Cry1Ac* DNA fragments of the positive control and the 19 transgenic lines were in accordance with the expected length (749 bp) (Figure 2). The anticipated 500-bp fragments produced in the PCR reactions from the positive control and the 19 transgenic plants with *npt*II primers are shown in Figure 3. The two exogenous genes were not detected in the non-transgenic control. The PCR analyses confirmed that the exogenous genes were effectively transferred into the transgenic lines as shown by successful amplification of the anticipated fragments. The results shown in Figures 2, 3 also indicate that the *Cry1Ac* and the *npt*II genes maintained a long-term presence after 6 years of field planting.

### Expression of the Cry1Ac protein in the 6-year-old plants

**Figure 4:**
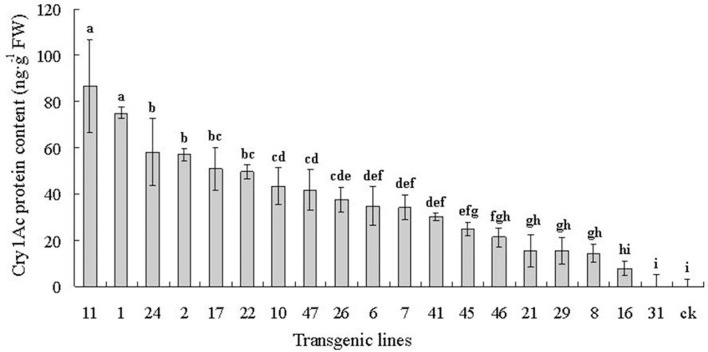
Cry1Ac protein content of 19 6-year-old transgenic lines detected using ELISA. Cry1Ac protein content are listed from highest to lowest. The toxic protein content varied greatly among the 19 6-year-old transgenic lines from 0.00 to 86.62 ng g^−1^ FW. Data are means ± SD of three replicates. Error bars represent the standard deviation of the mean. Different letters above the bars indicate a statistically significant difference between the transgenic lines at the *P* < 0.05 level according to Duncan's multiple range test.

The Cry1Ac protein content is reported in nanograms per gram of leaves in fresh leaf weight (FW) (Figure 4). Toxin levels are listed from highest to lowest. The toxic protein content varied greatly among the 19 6-year-old transgenic lines from 0.00 to 86.62 ng g^−1^ FW. Variance and multiple comparisons analysis showed that a significant difference existed between the different lines (ANOVA, *F* = 30.20, *P* < 0.0001). There were statistically significant differences among most of the transgenic lines at the *P* < 0.05 level (Duncan's multiple range test). Line 11 had the highest toxin content at 86.62 ng g^−1^ FW, whereas no toxin was detected in line 31 or in the control.

### Insect bioassay

#### Mortality rates measurement

The 3-year-corrected larvae mortalities of the *C. anachoreta* larvae are shown in Table 1. The corrected mortality rates differed between lines of different ages: 10 lines (52.6%) of the 1-year-old transgenic plants and 8 lines (42.1%) of the 2-year-old transgenic plants had *C. anachoreta* larval mortality rates exceeding 80%, whereas only five lines (26.3%) of the 6-year-old transgenic plants had *C. anachoreta* larval mortality rates exceeding 80%. The proportion of 6-year-old transgenic lines with over 80% larval mortality rates was clearly lower than that of the younger trees. Furthermore, 11 lines (lines 1, 6, 8, 10, 17, 22, 24, 29, 41, 46, and 47) showed lower insect resistance compared with the one- and 2-year-old saplings, occupying a percentage of 57.9%.

**Table 1:** Corrected mortality rates (%) of *C. anachoreta* and *L. dispar* for 1-, 2-, and 6-year-old plants of 19 transgenic lines.

**Transgenic lines**	***C. anachoreta***				***L. dispar***			
	**1-year-old**	**2-year-old**	**6-year-old**	**average**	**1-year-old**	**2-year-old**	**6-year-old**	**average**
1	100	100	79.8	93.3^a^	100	100	83.4	94.5^a^
2	80.0	75.5	82.0	79.2^abcd^	100	100	91.6	97.2^a^
6	100	85.1	77.3	87.5^ab^	100	85.0	58.4	81.1^a^
7	92.2	79.8	100	90.7^a^	100	100	87.5	95.8^a^
8	100	78.7	58.3	79.0^abcd^	96	100	62.5	86.2^a^
10	78.4	66.0	57.1	67.2^bcde^	100	80.0	91.6	90.5^a^
11	100	84.0	100	94.7^a^	100	100	79.1	93.0^a^
16	0.0	9.6	7.2	5.6^i^	39.7	29.0	37.5	35.4^b^
17	82.9	100	64.7	82.5^abc^	100	86.0	66.6	84.2^a^
21	96.0	100	100	98.7^a^	100	100	91.6	97.2^a^
22	56.8	59.6	35.6	50.7^efg^	100	81.0	85.8	88.9^a^
24	41.9	50.0	31.9	41.3^fg^	92.8	84.0	90.6	89.1^a^
26	35.2	25.5	39.9	33.5^gh^	95.5	82.0	90.0	89.2^a^
29	70.2	62.8	47.0	60.0^def^	100	100	93.9	98.0^a^
31	3.56	18.1	30.3	17.3^hi^	39.7	29.0	37.5	35.4^b^
41	82.0	100	69.7	83.9^ab^	90.2	71.0	91.6	84.3^a^
45	80.1	97.0	93.0	90.0^a^	100	100	87.5	95.8^a^
46	67.2	70.5	51.6	63.1^cde^	100	100	70.9	90.3^a^
47	76.7	95.0	66.5	79.4^abcde^	100	100	91.6	97.2^a^
Average	70.7^a^	71.4^a^	62.7^a^	−	92.3^a^	85.6^b^	78.4^c^	−

*Data are means of three replicates. Within the column, the numbers followed by the same letter are not significantly different (Duncan's multiple range test, at P < 0.05 level)*.

**Figure 5:**
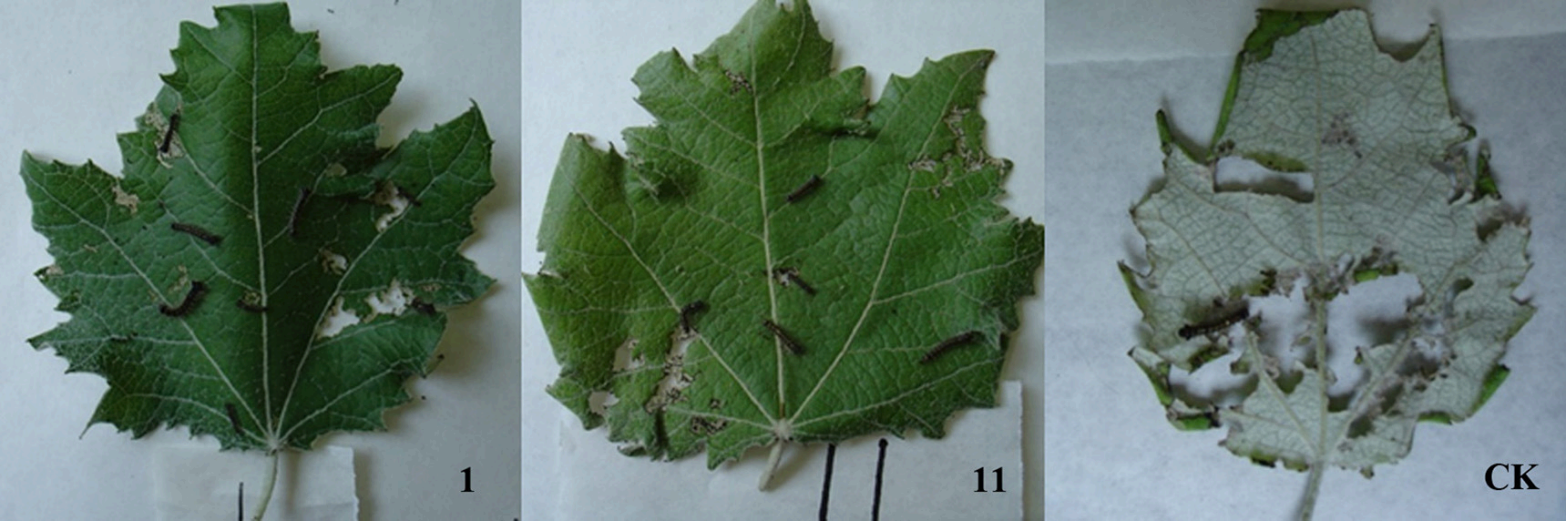
The situation of *L. dispar* larvae feeding on the leaves of three 1-year-old plants. **(1)** The situation of *L. dispar* larvae feeding on the leaf of transgenic line 1. (11) The situation of *L. dispar* larvae feeding on the leaf of transgenic line 1. (CK) The situation of *L. dispar* larvae feeding on the leaf of non-transgenic poplar. The *L. dispar* larvae feed more non-transgenic poplar leaf, but less transgenic line leaves.

The mortality rates of *L. dispar* exhibited the same trend. When the trees were 1-year-old saplings, 89.5% (17 lines except lines 16 and 31) showed high toxicity with mortality ≥ 80%. When the trees were 2-year-old saplings, 84.2% (16 lines except lines 16, 31, and 41) showed high toxicity with mortality ≥ 80%. For large 6-year-old trees, the proportion with mortality ≥ 80% decreased to 63.2% (12 lines). The situation of *L. dispar* larvae feeding on the leaves of three 1-year-old plants were shown in Figure 5. The *L. dispar* larvae feed more non-transgenic poplar leaf, but less transgenic line leaves.

From the above mortality comparison of 6-year-old large trees to 1- and 2-year-old saplings, we can see that in the first year or two after planting in the field, a high proportion of trees exhibited insect mortality greater than or equal to 80%. By contrast, in 6-year-old trees, the proportion of high resistance decreased and that of medium and low resistance increased. More than half of the transgenic lines had decreased toxicity to *C. anachoreta* and *L. dispar* insects in 6-year-old trees.

The average corrected mortality rates for the 1-, 2-, and 6-year-old transgenic lines of the two types of insect species are also shown in Table 1. The average corrected mortality rates of *C. anachoreta* ranged from 5.6 to 98.7%, whereas the average corrected mortality rates of *L. dispar* ranged from 35.4 to 97.2%. The mortality differed significantly between different transgenic lines (ANOVA, *F* = 18.36, *P* < 0.0001 for *C. anachoreta, F* = 13.00, *P* < 0.0001 for *L. dispar*). During the experimental period, eight transgenic lines exhibited average *C. anachoreta* larval mortality rates exceeding 80%; among them, lines 1, 7, 11, 21, and 45 showed no significant difference at the *P* < 0.05 level (Duncan's multiple range test). There were eight lines that exhibited mortality rates of 40–80% and three lines that exhibited mortality rates less than 40%. *Lymantria dispar* was found to be more susceptible to the transgene compared with *C. anachoreta*. In 3 years, 17 lines had over 80% average larval mortality rates, and only lines 16 and 31 had low larval mortality rates (with 35.4%). There was no difference between lines 16 and 31, but they both showed significant differences compared to the other 17 lines (Duncan's multiple range test, *P* < 0.05). The four lines of single copy detected by Southern blot were all higher insect resistances.

Insecticidal effect of 1- and 6-year-old trees on different instar larvae.

**Figure 6:**
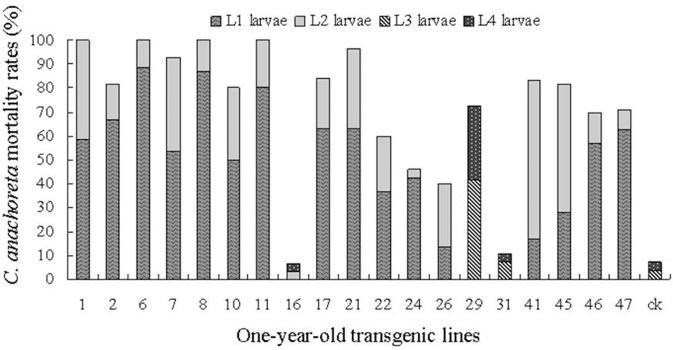
*C. anachoreta* death distribution in different developmental stages of 1-year-old trees. Data are means of three replicates. This mortality is not corrected mortality. The larvae that fed on the leaves of 1-year-old trees were killed mostly during L_1_ and L_2_. Few larvae survived to L_3_ and L_4_, and some larvae exhibited arrested body development.

**Figure 7:**
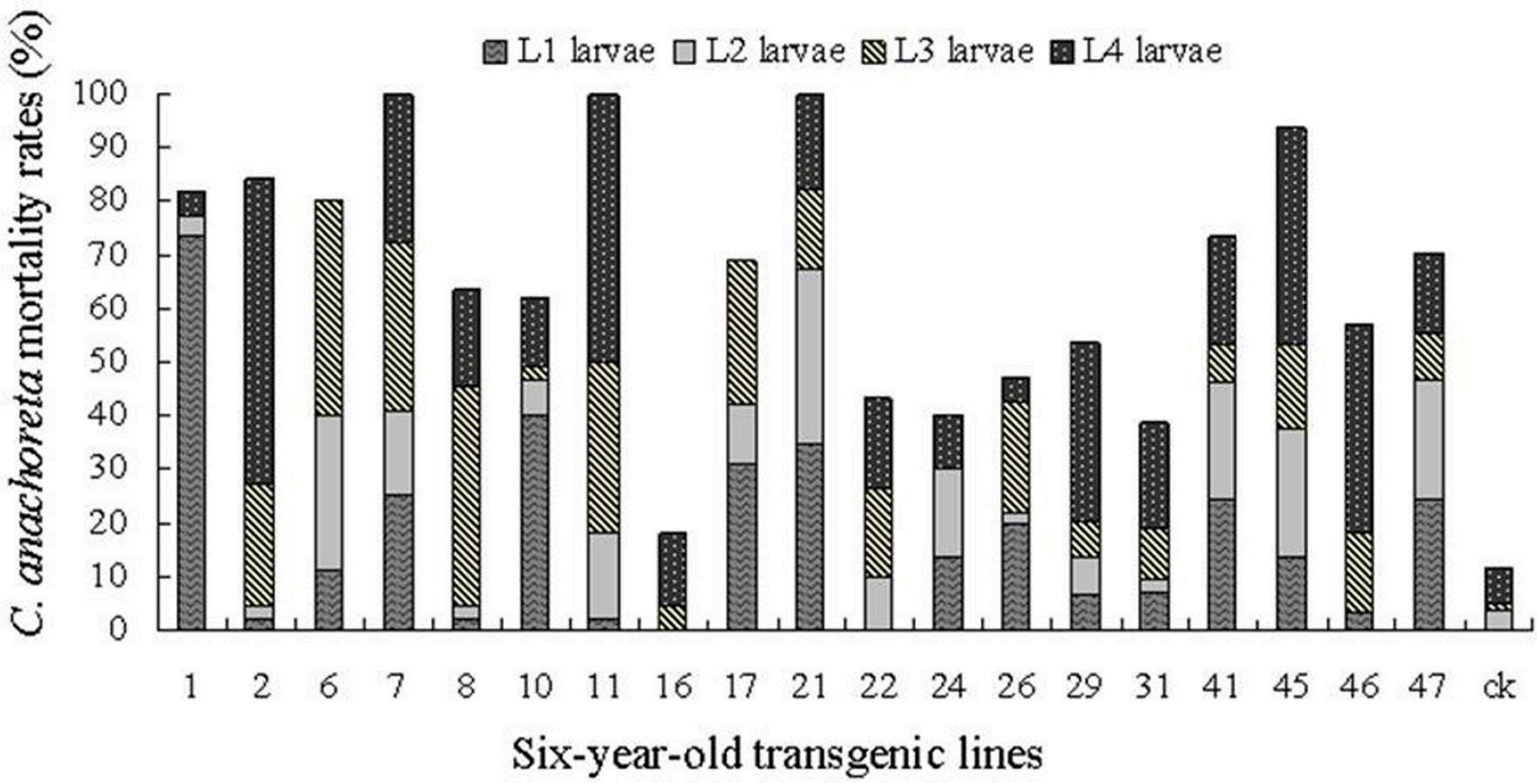
*C. anachoreta* death distribution in different developmental stages of 6-year-old trees. Data are means of three replicates. This mortality is not corrected mortality. Larval death was observed at different stages of 6-year-old trees. The proportion of larvae that died in the L_3_ and L_4_ stages increased.

Figures 6, 7 show the death distribution of *C. anachoreta* at different developmental stages (L_1_, L_2_, L_3_, and L_4_). This mortality is not corrected mortality. Thus, the mortality of the control plant is presented in the bar with 7.4 and 11.9% of the 1- and 6-year-old trees separately. Figure 6 shows that the larvae that fed on the leaves of 1-year-old trees were killed mostly during L_1_ and L_2_. Few larvae survived to L_3_ and L_4_, and some larvae exhibited arrested body development. Larval death was observed at different stages of 6-year-old tree development and was mainly distributed in L_3_ and L_4_ (Figure 7). The proportion of larvae that died in the later stages increased. Although many 6-year-old transgenic lines had high larval mortality, the death hysteresis phenomenon also appeared. Insects can survive to a high developmental stage, and damage to the insect caused by leaves with toxic substance decreased over time. Thus, the insect resistance of the 6-year-old tree exhibited a downward trend.

### Correlation analyses

#### Correlation analysis among tree ages, larval mortality rates, and different insects

We analyzed the correlations of different larval mortality rates among different years (Table 2). For *L. dispar*, the correlation coefficient between 1- and 2-year-old trees was 0.932, that between 1- and 6-year-old trees was 0.777, and that between 2- and 6-year-old trees was 0.710. It is clear that the correlations between 1- and 6-year-old trees and between 2- and 6-year-old trees declined*. Clostera anachoreta* and exhibited the same trends: the correlation coefficient between 1- and 2-year-old trees was 0.906, that between 1- and 6-year-old trees was 0.844, and that between 2- and 6-year-old trees was 0.814. The results revealed significant correlations between the trees with different ages (*P* < 0.01). The correlation between 1- and 2-year-old trees was higher than that between 1- and 6-year-old trees and that between 2- and 6-year-old trees. These findings indicate that the transgenic lines retained insect resistance in the first 2 years, but this resistance decreased over time.

**Table 2:** Correlation coefficients of different larval mortality rates of 19 transgenic lines among different years.

**Tree age**	**Insect species**	**1-year-old**		**2-year-old**		**6-year-old**	
		***C. anachoreta***	***L. dispar***	***C. anachoreta***	***L. dispar***	***C. anachoreta***	***L. dispar***
1-year-old	*C. anachoreta*	1.000	0.817^**^	0.906^**^	0.827^**^	0.844^**^	0.487^*^
	*L. dispar*		1.000	0.732^**^	0.932^**^	0.620^**^	0.777^**^
2-year-old	*C. anachoreta*			1.000	0.733^**^	0.814^**^	0.488^*^
	*L. dispar*				1.000	0.692^**^	0.710^**^
6-year-old	*C. anachoreta*					1.000	0.450
	*L. dispar*						1.000

* Significant correlation (α = 0.05, r_α_ = 0.444, n = 19);

** *Highly significant correlation (α = 0.01, r_α_ = 0.561, n = 19). Underlined correlation coefficients were used in Table 2*.

The English in this document has been checked by at least two professional editors, both native speakers of English. For a certificate, please see: http://www.textcheck.com/certificate/gI4qbH

Correlation analyses between mortality rates and Cry1Ac protein expression of 6-year-old trees.

**Figure 8:**
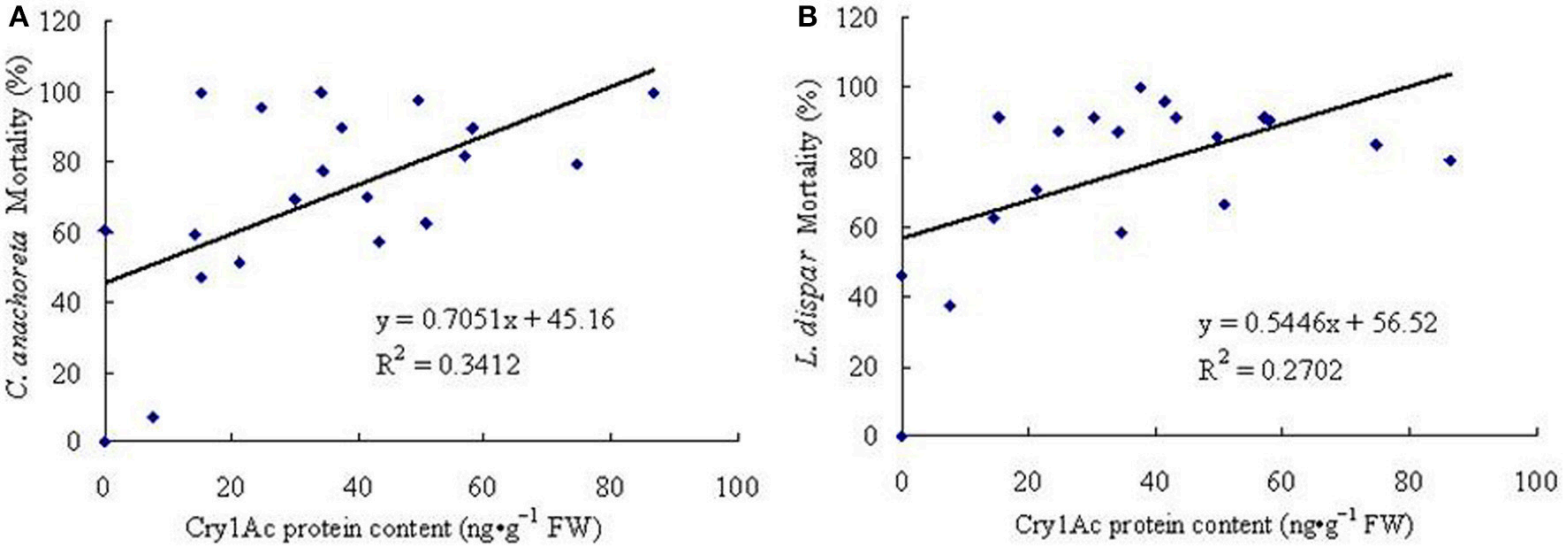
Correlations between insect mortality rates and Cry1Ac protein content of 19 6-year-old transgenic lines. **(A)** Correlation between *C. anachoreta* mortality rate and Cry1Ac protein content of 19 6-year-old transgenic lines. **(B)** Correlation between *L. dispar* mortality rate and Cry1Ac protein content of 19 6-year-old transgenic lines. Each figure uses the toxin protein content of 19 transgenic lines (abscissa) and the mortality rate of insect larvae (ordinate) to make a scatterplot. In the end, a fitted linear equation was obtained according to the coordinates of points in the figure.

ELISA conducted on the leaves of 6-year old trees revealed Cry1Ac protein expression. We also conducted a correlation analysis of larval mortality rates and Cry1Ac protein expression. For *C. anachoreta* (Figure 8A), the correlation coefficient between mortality rates and Cry1Ac protein expression was *r* = 0.584, with high correlation at α = 0.01 (*r*_α_ = 0.561, *n* = 19). For *L. dispar* (Figure 8B), the correlation coefficient was *r* = 0.520, with high correlation at α = 0.05 (*r*_α_ = 0.444, *n* = 19). The results of this study showed that Cry1Ac protein expression is significantly correlated with the insecticidal activity of transgenic plants.

## Discussion

Poplars are perennial woody plants propagated through cutting, grafting, and other asexual propagation methods. We determined whether the exogenous genes in transgenic poplars persisted during multiple asexual propagations and through years of field cultivation. Few studies have reported on the long-term efficacy of transformed pest-resistant poplars (Hu et al., 2001; Yang et al., 2005; Ren et al., 2017). This study presented a continuous and dynamic view of the insect resistance changes of different transgenic lines under field conditions. In our study, PCR analysis of 6-year-old *P. tomentosa* proved that the exogenous genes *Cry1Ac* and *npt*II had a stable presence in the transgenic *P. tomentosa* genomes, with no loss of any exogenous gene. Hu et al. (2004) detected 7-year-old transgenic *P. nigra* with the *Bt* gene. Mendelian segregation of the *Bt* gene was observed in the progeny of transgenic *P. nigra* (clone 153) crossed with the control. The ratio of gene segregation in the autosomal chromosomes was consistent with a 1:1 ratio, but no further study or discussion was conducted on gene expression and insect resistance changes in that study.

The insect-feeding experiment on transgenic lines of different ages in this study demonstrated significant differences in the mortality rates of the two types of insect species between different ages. Insect resistance of the transgenic lines changed with increasing tree age. The proportion of 6-year-old transgenic lines with over 80% larval mortality rates was clearly lower than those of the younger trees, whereas the proportion of 6-year-old transgenic lines with larval mortality rates less than 80% greatly increased. These observations indicate that the insect resistance of some highly toxic plants decreases over time, whereas some plants still retain high toxicity with increasing tree age. The correlations of the mortality rates between the different insect species and between the different ages were significant. The correlation coefficient between the 1- and 2-year-old trees was higher than that between the 1- and 6-year-old trees. This finding further proves that the insect resistance of some lines decreased with increasing tree age. The larval mortality rates were significantly correlated with the amount of Bt protein expression, as indicated by ELISA. The ELISA analyses helped predict the insect resistance of transgenic plants. Combining toxin protein ELISA analyses with insect bioassays will facilitate the efficient selection of transgenic plants with different levels of insect resistance.

The decrease in the insect resistance of some transgenic plants may have different causes. Previous studies have shown that changes in the external environment (Meyer and Heidmann, 1994), the growth and development of plants, sexual hybridization (Scheid et al., 1991), grafting (van Slogteren et al., 1984), and other factors can change the degree of DNA methylation and change the expression of exogenous genes. The number of deactivated transgenic lines also increases with stronger light and higher temperatures (Scheid et al., 1991). Meyer and Heidmann (1994) found that transplanting a transgenic line to the field increases the deactivation of exogenous genes. Trees have special biological characteristics, such as long growth periods, large tree bodies, and seasonal dormancy. Exogenous gene expression in genetically modified trees has its particularity, and little research has been done on the changes in exogenous gene expression over time. Chen et al. (2000) found the expression of Bt toxin protein in *Bt* transgenic cotton decreased with advance of development. In our study, insect resistance decreased in some transgenic lines after many years of field planting. The direct reason should be the change of Bt protein expression level. This may be attributed to the changes in metabolic processes when poplars mature compared with the corresponding changes in metabolic processes in the juvenile stages (Fladung and Ewald, 2006), the influence of the external environment (Meyer and Heidmann, 1994), or the degree of DNA methylation (Finnegan and McElroy, 1994; Li et al., 1998). With the growth of the tree, the metabolism of perennial tree may not be as strong as the seedling stage, and the level of exogenous gene expression may decrease. The variations in insect resistance of different lines were different, which may be related to the degree of DNA methylation. The investigation of Fladung and Ewald (2006) was also conducted in different years under the influence of environmental factors, such as climate conditions that may have caused changes in the transgenic insect-resistant poplars. Additionally, exogenous genes located at different chromosomal positions could be affected by their own gene expression changes, resulting in exogenous gene transcription and expression decline in some transgenic line (Mlynarova et al., 1994). The results of our study are only preliminary. Further study and long-term observation are needed on the resistance change rules and the mechanisms of mature transgenic trees.

The application of transgenic insect-resistant lines can dramatically alter the insect resistance of plants. However, this approach has some potential issues. Aside from environmental safety, insect tolerance of insect-resistant plants is a problem that cannot be neglected. Thus, appropriate measures to limit the evolution of insect tolerance must be considered. Studies have shown that transgenic lines have various levels of resistance after genetic transformation (Génissel et al., 2003). However, previous studies have often focused on the selection and utilization of transgenic lines with high resistance. Although such an approach can control and kill insect pests effectively, the genetic diversity and stability of the forest will be reduced as a result of using a single high insect-resistance line and will easily lead to the quick appearance and evolution of pest populations with tolerance. Therefore, using a single high-resistance line in afforestation is not necessarily the best method for controlling pests with regard to ecological environmental safety and sustainable pest control. We must consider controlling pest populations based on the level of economic harm rather than by killing them. Thus, exploring and designing mixed afforestation modes by using genetically modified line with high and medium insect resistance is more conducive to maintaining the variety and stability of transgenic line, to decreasing the occurrence of tolerance in insect populations, and to further preventing insect tolerance (Andow and Zwahlen, 2006; Hu et al., 2010).

## Author contributions

YR analyzed the data and edited the manuscript. GW and XL analyzed the data, and wrote the manuscript. LL and JZ conducted the experiments and analyzed the data. JW designed the experiments, analyzed the data and edited the manuscript. MY designed the experiments and edited the manuscript.

### Conflict of interest statement

The authors declare that the research was conducted in the absence of any commercial or financial relationships that could be construed as a potential conflict of interest.
